# Restoration of reproductive capacity in a male patient with congenital adrenal hyperplasia and bilateral testicular adrenal rest tumors (TARTs) after six months of glucocorticoid intensification: A case report

**DOI:** 10.1097/MD.0000000000036061

**Published:** 2023-12-08

**Authors:** Jihan Ahmad, Adnan Ahmad, Lama Hadid

**Affiliations:** a Department of Endocrinology, Al-Assad University Hospital, Damascus University, Damascus, Syria; b Department of Urology, Al-Assad University Hospital, Damascus University, Damascus, Syria.

**Keywords:** case report, congenital adrenal hyperplasia, infertility, oligospermia, testicular adrenal rest tumors

## Abstract

**Rationale:**

Congenital adrenal hyperplasia (CAH) is considered one of the most common inherited disorders. In about more than 95% of all CAH cases, the deficient enzyme is 21-hydroxylase. Infertility is an important complication of this disease, and although this topic has been studied more frequently in females, cases, and literature reviews of the causes of infertility in male patients are constantly increasing.

**Patient concerns:**

A 28 old male with congenital adrenal hyperplasia (we assume to be a nonclassical type) presented to our institution with infertility and suspected bilateral testicular masses after 4 years of stopping dexamethasone.

**Diagnosis:**

Testicular adrenal rest tumors.

**Interventions:**

Dexamethasone was reapplied in a supraphysiologic dose (1.5 mg before bedtime) with periodic monitoring of the patient.

**Outcomes:**

Treatment with supraphysiologic dose of dexamethasone led to regression of these tumors and significant improvement in sperm count, resulting in being capable of having a child.

**Lessons:**

There are many suspected causes of reduced male fertility in male CAH patients and the presence of testicular adrenal rest tumors is the main cause of infertility in this population. These benign tumors are believed to arise from ectopic adrenal cells in the testes, that grow under adrenocorticotropic hormone stimulation in poorly controlled patients. Annual scrotal ultrasound is recommended in all males with CAH for detection and treatment of these tumors as early as possible before they cause permanent damage to the seminiferous tubules and irreversible infertility.

## 1. Introduction

Adrenogenital syndrome, or what is called congenital adrenal hyperplasia (CAH), is considered the most common congenital adrenal disease, and it includes a group of enzyme deficiencies in the adrenal steroidogenesis pathway. In about more than 95% of all CAH cases, the deficient enzyme is 21-hydroxylase. This enzyme is responsible for converting 17-hydroxyprogesterone to 11-deoxycortisol (precursor for cortisol), and thus its deficiency causes decrease synthesis of cortisol in addition to a deficiency in the synthesis of mineralocorticoids (aldosterone) in severe cases.^[1]^ Clinical manifestations of this syndrome depend on the degree of enzymatic deficiency and are due to a combination of cortisol and aldosterone deficiency, in addition to androgen excess (caused by the accumulation of metabolites or precursors of steroids in the androgen synthesis pathway).

The clinical spectrum of this disease includes the following:

1. The classical type: includes the salt-wasting form and the non- salt-wasting form, depending on the degree of aldosterone deficiency. These forms are usually diagnosed during early childhood.
2. The nonclassical type: in which there is 20% to 50% 21 hydroxylase enzyme activity. This type presents later in life with signs of hyperandrogenism.

The incidence of the classical type ranges from 1:12,000 to 1:20,000 live births worldwide and of the nonclassical type was estimated to be 0.1% worldwide and even more common in specific populations.^[2,3]^

Infertility is one of the most important complications of this syndrome, which has been studied more widely in females than in males. Several studies documented lower fertility rates in men with CAH compared to men in the general population.^[4,5]^ There are many causes of infertility in male patients with CAH:

① Testicular adrenal rest tumors (TARTs) which are thought to arise from aberrant adrenal cells in the testes, are considered the most important cause of infertility in these patients. ② Hypogonadotrophic hypogonadism that can be induced by negative feedback of excess androgens. ③ Dysfunction of Sertoli and Leydig cells (testicular failure). ④ Other factors such as excessive treatment with glucocorticoids, and elevated body mass index.^[6]^

The pathogenesis of TARTs is not fully understood, but they are believed to be derived from ectopic adrenal cells that descend with the testes during embryological development and grow under the influence of high concentrations of adrenocorticotropic hormone (ACTH), which is usually seen in cases of poor control of the disease.^[7,8]^ Growth of TARTs may cause infertility by compression of the normal testicular tissue and the seminiferous tubules, which leads to obstruction of semen outflow. It may also impair blood flow to the normal surrounding testicular tissue. In addition to mechanical obstruction, some studies found a possible toxic paracrine effect of local steroids secreted by the tumor cells.^[9]^

## 2. Case presentation

A 28 old male presented to our hospital’s urology clinic with infertility and suspected bilateral testicular masses. The patient was diagnosed with congenital adrenal hyperplasia at 4 years of age after developing pubic hair (premature adrenarche) which was treated with dexamethasone.

### 2.1. Personal information and history

The patient was a nonsmoker, married for 5 years, and has a 4-year-old son. The patient’s family history includes a sister with congenital adrenal hyperplasia diagnosed when she was a newborn with ambiguous genitalia.

*Drug history*: Dexamethasone 0.5 mg before bedtime over the course of his life, but he admitted that he stopped taking the drug 4 years ago by himself.

*Surgical history*: repair of a right inguinal hernia in 2012.

### 2.2. Clinical findings

His vital signs were: blood pressure in the sitting position (100/70) without orthostatic hypotension, heart rate (76/m), and respiratory rate (18/m). His weight (70 kg), height(148 cm), and body mass index (32 kg/m^2^). On clinical examination, the testes appeared nodular on palpation with no pain or any abnormal changes in the scrotal skin. He did not show any clinical cushioned features. The rest of his clinical examination was normal.

### 2.3. Laboratory and imaging results

The patient had done 2 semen analyses and both revealed severe oligozoospermia (the results in detail): volume (1.5 mL), viscosity (normal), pH (8.5), sperm count (2.4 × 10^6^/mL), the count in the second test (2.1 × 10^**6**^/mL), normal forms (75%), direct motility (30%), motility after 1 hour (25%).

**Figure 1.**
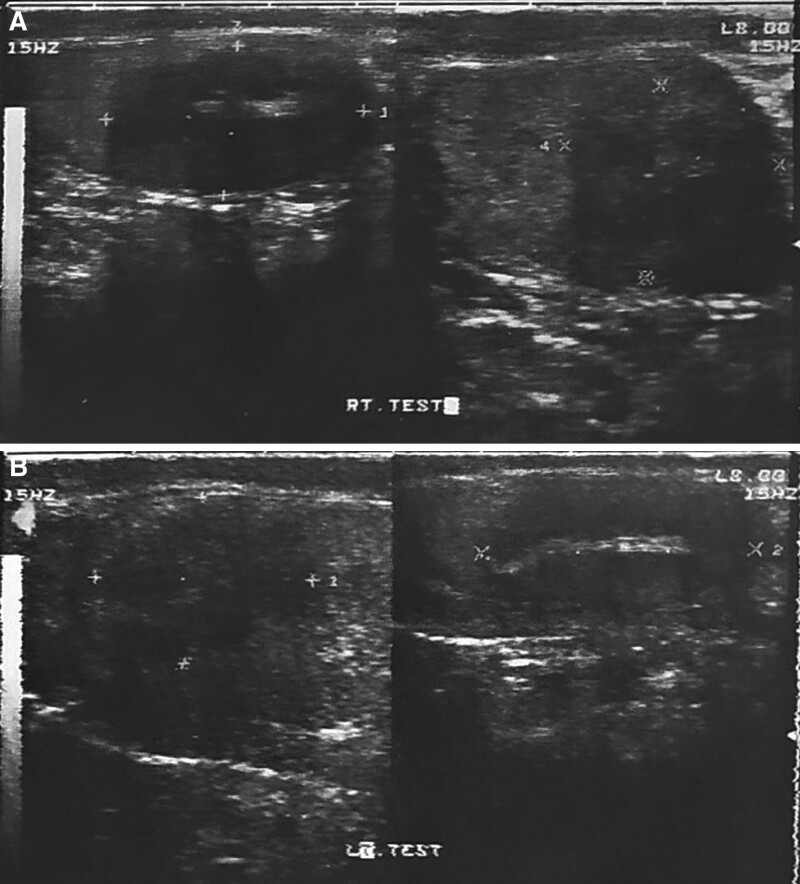
Gray-scale ultrasound images of the right (A) and left testes (B). Both testes are mostly occupied by hypoechoic lesions with clear irregular margins and linear increased echogenicity in the central area.

A scrotal ultrasound was subsequently performed and showed bilateral hypoechoic masses with irregular margins and linear increased echogenicity in the central area in both testis, the right-sided mass measures 30 * 19 * 18 mm and it occupies approximately 75% of the right testes. The left mass measures 31 * 18 * 18 mm and it also occupies most of the left testis (Fig. 1). Color Doppler showed disorganized low color flow (hypovascularity) within the lesions. Tumor markers lactate dehydrogenase, beta-HCG, and alpha-fetoprotein were normal. An endocrine evaluation revealed markedly elevated ACTH levels (981 pg/mL, [7.2–63.3] pg/mL) with low morning 8 AM cortisol (1.93 µg/dL, [6.02–18.4] µg/dL), low luteinizing hormone (0.10 mIU/mL, [1.7–8.6] mIU/mL), normal total testosterone (3.57 ng/mL, (2.8–8) ng/mL), and normal dehydroepiandrosterone sulfate (327 µg/dL, [160–449] µg/dL).Blood glucose and electrolytes were normal.

### 2.4. Management

Treatment with supraphysiologic doses of dexamethasone was applied (1.5 mg before bedtime) and we reevaluate the patient after 5 months. The scrotal ultrasound revealed normal testicles with absence of previously described masses. His new semen analysis demonstrated significant improvement in sperm count from 2.4 million per mL to 18 million per mL. We also perform an endocrine reevaluation which showed a decrease in ACTH levels (5.19 pg/mL,(7.2–63.3) pg/mL), and a controlled 17 hydroxy-progesterone (0.66 ng/mL [0.2–1.36] ng/mL). Blood glucose and electrolytes remains normal. We then decreased the dose of dexamethasone to 1 mg before bedtime. A month after this evaluation the patient stated that his wife turns out to be pregnant.

## 3. Discussion

Lack of cortisol synthesis in the congenital adrenal hyperplasia results in remarkably elevated levels of ACTH which in turn causes hyperstimulation of the adrenal glands. Furthermore, testicular adrenal rest tumors may develop in males with CAH due to overstimulation of aberrant adrenal cells within the testes by high concentrations of plasma ACTH. These benign tumors tend to be bilateral, originating near the mediastinum testis (rete testes), and can be painful when reaches large sizes.^[10]^ They typically appear hypoechoic and often do not demonstrate any internal vascularity on Doppler ultrasound.^[11,12]^ The reported prevalence of TARTs was 34% in the largest series to date from France which includes 219 male CAH patients.^[5]^ However, there are widely variable prevalence rates of TARTs which in some studies reach up to 94%.^[13]^ This discrepancy may be due to the number of the sample or to the age of the included patients. Low sperm count was found in 66 % of patients from the previously mentioned French survey. Severe oligospermia and azoospermia were found in 42% of patients and were more prevalent in men with TARTs (70%) compared to men with normal testes.^[5]^ In another study conducted in London on 50 patients, the male patients with severe oligospermia (11 out of 23) were more likely to have a TART on ultrasound (81.8 %).^[14]^ Most of the published studies involved patients with the classic type of congenital adrenal hyperplasia and rarely includes the nonclassical type. Therefore, the exact incidence of TARTs in patients with nonclassical congenital adrenal hyperplasia is unknown. In this case, our patient was diagnosed with CAH when he was 4 years old based on developing adrenarche which supported the diagnosis of nonclassical type of congenital adrenal hyperplasia. The patient’s short stature indicates poor adherence to treatment during the entire period after being diagnosed, which was a result of excessive androgens that cause premature epiphyseal closure. Optimization and intensification of glucocorticoid therapy are essential in the management of testicular adrenal rest tumors, which can suppress ACTH, reduce TARTs size and improve testicular function. This approach gives the best results if these tumors are treated in the early stages, rather than the later stages of TARTs development which then may be ineffective.^[10,15]^ Surgical removal of TARTs may be required is some patients for pain relief, but can cause further testicular damage.^[16]^ Although our patient visited our clinic 4 years after complete cessation of steroids, treatment with supraphysiologic dose of dexamethasone led to disappearance of testicular masses, significant increase in sperm count, and restoration of fertility after a relatively short period of time.

## 4. Conclusion

Testicular adrenal rest tumors TARTs is the leading cause of infertility in male patients with congenital adrenal hyperplasia. It was found in most cases of severe oligospermia. It is recommended to perform a scrotal ultrasound in all CAH patients for detecting TARTs, starting in adolescence and annually afterward, regardless of the patient’s disease control, as some authors have found these tumors in some of their well-controlled CAH patients, suggesting the presence of growth factors other than ACTH that may contribute to the growth of these tumors.^[17]^ Response to steroids intensification depends on the stage of these tumors, therefore early recognition and prompt initiation of treatment of TARTs is essential for preserving gonadal functions and avoiding permanent testicular damage.

## Author contributions

**Conceptualization:** Jihan Ahmad, Adnan Ahmad, Lama Hadid.

**Data curation:** Jihan Ahmad, Adnan Ahmad, Lama Hadid.

**Investigation:** Adnan Ahmad, Lama Hadid.

**Project administration:** Adnan Ahmad, Lama Hadid.

**Supervision:** Jihan Ahmad, Adnan Ahmad, Lama Hadid.

**Validation:** Adnan Ahmad, Lama Hadid.

**Visualization:** Adnan Ahmad, Lama Hadid.

**Writing – original draft:** Jihan Ahmad.

**Writing – review & editing:** Adnan Ahmad, Lama Hadid.
